# Collective Migration of Lens Epithelial Cell Induced by Differential Microscale Groove Patterns

**DOI:** 10.3390/jfb8030034

**Published:** 2017-08-09

**Authors:** Chunga Kwon, Youngjun Kim, Hojeong Jeon

**Affiliations:** 1 Korea Institute of Science and Technology Europe (KIST-Europe) Forschungsgesellschaft mbH, Campus E 7 1, 66123 Saarbrücken, Germany; c.kwon@kist-europe.de; 2 Center for Biomaterials, Biomedical Research Institute, Korea Institute of Science and Technology (KIST), Hwarangno 14-gil 5, Seongbuk-gu, Seoul 136-791, Korea; 3 Department of Biomedical Engineering, University of Science and Technology (UST), Daejeon 34113, Korea

**Keywords:** polydimethylsiloxane (PDMS), micro-patterns, human lens epithelial cells (B-3), microgroove patterns, posterior capsular opacification (PCO)

## Abstract

Herein, a micro-patterned cell adhesive surface is prepared for the future design of medical devices. One-dimensional polydimethylsiloxane (PDMS) micro patterns were prepared by a photolithography process. We investigated the effect of microscale topographical patterned surfaces on decreasing the collective cell migration rate. PDMS substrates were prepared through soft lithography using Si molds fabricated by photolithography. Afterwards, we observed the collective cell migration of human lens epithelial cells (B-3) on various groove/ridge patterns and evaluated the migration rate to determine the pattern most effective in slowing down the cell sheet spreading speed. Microgroove patterns were variable, with widths of 3, 5, and 10 µm. After the seeding, time-lapse images were taken under controlled cell culturing conditions. Cell sheet borders were drawn in order to assess collective migration rate. Our experiments revealed that the topographical patterned surfaces could be applied to intraocular lenses to prevent or slow the development of posterior capsular opacification (PCO) by delaying the growth and spread of human lens epithelial cells.

## 1. Introduction

Cataract is one of the most common diseases of elderly people, and is responsible for considerable medical costs to patients. About 40% of blindness is caused by cataracts. According to the estimates of the Eye Diseases Prevalence Research Group, the number of persons who have cataract is expected increase up to 30.1 million by 2020 [1,2]. 

Cataract can be treated with simple and relatively safe surgery. The most commonly used surgery is phacoemulsification that uses an ultrasound probe to emulsify cataract. After the removal of the clouded lens, an intraocular lens (IOL) is introduced in place of the vacated lens. A person with pseudophakia might experience a secondary loss of vision caused by posterior capsular opacification (PCO) that is caused by residual lens epithelial cells (LECs) left behind during cataract surgery. The remaining LECs migrate to the posterior capsule and undergo proliferation, collagen deposition, and epithelial–mesenchymal transition (EMT), which eventually leads to cloudiness in the capsule. The incidence of PCO is reported to be 20–40% 2 to 5 years after the cataract surgery, with higher incidence in infants and young children [3,4]. Once the PCO occurs, the patients are subjected to Nd:YAG (Neodymium-doped Yttrium Aluminum Garnet) laser capsulotomy to treat the symptoms. The laser surgery claims to be safe and relatively non-invasive, but it is reported to have severe side effects, such as long-term increases in intraocular pressure (IOP), cystoid macular edema, retinal detachment, iris hemorrhage, etc. In addition, the treatment is not only known to cast a considerable burden on the national health care system, but is unavailable in developing countries [5,6,7]. Therefore, the prevention of PCO is cost-beneficial and can allow patients to avoid additional surgery.

Currently there are various strategies to prevent PCO, such as surgical techniques, the use of advanced intraocular lens designs and materials, and therapeutic methods. Surgical technique aims for physical removal of remaining LECs during cataract surgery. Ophthalmologists would consider using specially-designed IOLs which have both material and physical properties of reducing LEC migration rate or proliferation. During surgery, therapeutic agents are used to destroy remaining LECs. Therapeutic agents can be injected directly in the anterior chamber, applied through irrigating solution, or can be impregnated in the IOL [8].

A simple design change of the IOL could reduce the incidence of PCO. Nishi et al. have shown a low incidence of PCO in sharp-edged IOL compared with round-edged IOL as a result of reduced migration of LECs toward posterior capsule. Sharp-edged IOLs formed capsular bends at the edges of the IOLs, thus inhibiting LECs from migrating to posterior areas [9]. Regarding the IOL material, hydrophobic materials were normally chosen as cells do not attach and proliferate on the hydrophobic surface. As a result, hydrophobic acrylic IOLs are reported to have lower capsulotomy rate than hydrophilic IOLs [10]. Most of the IOLs are made of hydrophobic materials that make it difficult for the cells to attach on the hydrophobic surface at the beginning. However, time passes and the induction of ECM releases from cells and non-selective serum protein start to cover the surface of the IOL, making the environment more favorable for the cell adhesion that causes the initial PCO. Despite these discoveries, PCO remains the most common complication of cataract surgery [11].

It is well known that micro- and nano-surface topography affects cell behavior such as cell morphology, migration, proliferation, differentiation, etc. [12,13,14]. In this study, we attempted to control the migration of LECs at cell–material interfaces by employing microscale surface topography. Since epithelial cells migrate, affecting adjacent cells (unlike mesenchymal cells), collective cell movement is mainly assessed [15]. There are many reports about the roles of topographical patterned surfaces in directing cell migration, a phenomenon called contact guidance. Teixeira et al. reported that human corneal epithelial cells responded to micro- and nanostructure cues, elongating and aligning along patterns [16]. Lawrence et al. has shown that micropatterned substrates have a guiding effect on collectively migrating cells, directing the cells to migrate along the long axis of the pattern, while the migration rate of cell sheets moving perpendicular to the feature significantly decreased [17]. 

Here, we observed human lens epithelial cells (B-3) on various groove/ridge patterns in the micrometer length scale and calculated respective collective cell migration rate to determine the most effective pattern in slowing down the cell sheet spreading speed. After cell seeding, time-lapse images were taken under the controlled cell culturing condition, and cell sheet borders were drawn in order to assess collective migration rate. Our experiments revealed that topographical patterned surfaces can be applied to intraocular lenses to prevent or slow the development of PCO by delaying human lens epithelial cells’ growth and spreading.

## 2. Materials and Methods

### 2.1. Designs of Patterns

The pattern used for the experiment had a non-patterned, flat area that worked as a seeding point in the middle. The microgroove pattern that was formed by repetitive groove and ridge units surrounded this area. The patterns varied in the width of ridge and groove; six types of patterns were used overall, including non-patterned flat surface as control, 3 µm/5 µm/10 µm width of ridges with 5 µm width of grooves and 3 µm/10 µm width of grooves with 5 µm width of ridges, while every pattern had the same height of 5 µm.

### 2.2. Fabrication of Patterned Substrates

**Figure 1:**
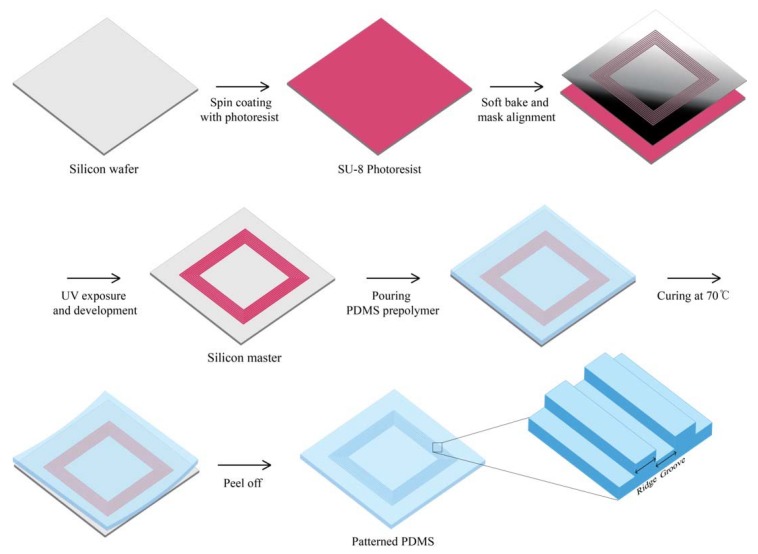
Schematic illustration of the fabrication process of patterned polydimethylsiloxane (PDMS). The master pattern of PDMS mold process using SU-8 photoresist and subsequent generation of the PDMS replication stamp.

The silicon master substrate was fabricated by UV lithography process. Negative photoresist (SU-8 3010 resist, MicroChem, Westborough, MA, USA) with thinner was coated on 4-inch silicon wafer by spin-coater at spin-speed of 4000 rpm for 45 s for 5 µm thickness. After a soft bake step for 2 min at 95 °C, the wafer was exposed to UV light (MA6 Aligner2, INRF, Irvine, CA, USA, UV Source 365 nm) under 100 mJ/cm^2^. During the developing process, the SU-8 layer that had not been exposed to UV light was dissolved in the developer, and the patterns were manufactured. Post-exposure bake was performed at 65 °C for 1 min and then at 95 °C for 1 min. The master substrate was silanized with 10 µL of trichloro(1H,1H,2H,2H)-perfluorooctylsilane (448931, Sigma-Aldrich, Saint Louis, MO, USA) vapor overnight under vacuum to make the template easier for the curing of polydimethylsiloxane (PDMS). To fabricate the PDMS molds with microscale topographical patterns, PDMS (Sylgard 184, Dow-Corning, Midland, MI, USA) was poured on the master surface, cured at 70 °C for 2 h, and peeled off (Figure 1). Then, the molds were immersed in high-purity ethanol and kept in a clean bench with UV light on for at least 2 h. The sterilized PDMS molds were coated with fibronectin (Bovine, Sigma-Aldrich, St. Louis, MO, USA) before cell seeding.

### 2.3. Cell Culture, Migration Monitoring, and Proliferation Assay 

**Figure 2:**
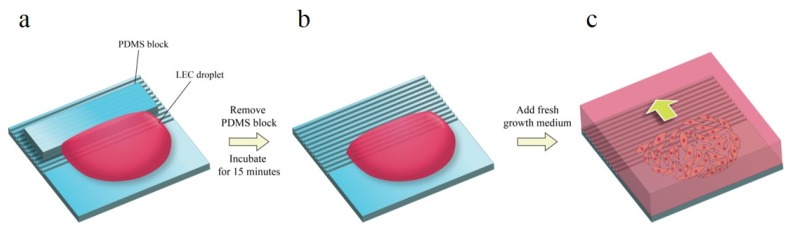
(**a**) Cell seeding procedure. PDMS block is covered on the patterned surface to prevent from the cell seeding point. (**b**) After PDMS block is removed, the cells are placed on the pattern for 15 min for cell adhesion. (**c**) The cell migration rates were measured from starting point every 24 h.

Human lens epithelial cell line B-3 (ATCC, Manassas, WV, USA) was cultured in minimum essential medium (Gibco, Thermo Fisher Scientific, Waltham, MA, USA) supplemented with 20% fetal bovine serum (FBS, Welgene, Gyeongsan, Korea) and 1% Antibiotic-Antimycotic (Gibco, Thermo Fisher Scientific, Waltham, Massachusetts, USA) at 37 °C, 5% CO_2_, and 90% humidity. A 20 µL droplet of cell suspension of 7 × 10^3^ cells was loaded on a fibronectin-coated pattern. In order to make the advancing outlines of the cell sheet linear, uncoated PDMS blocks were positioned upon the pattern (Figure 2). After 15 min of the incubation of seeded cells, fresh medium was added. Phase contrast images were taken right after adding growth medium via optical microscopy, and the cells were incubated for 24 h for the next imaging. The images were acquired from four positions of one pattern. 

Outlines of the borders were drawn manually. Using Image J, the area between day 0 and day 4 was evaluated.

(1) $$\mathrm{Average}\;\mathrm{migration}\;\mathrm{distance}=\frac{\mathrm{Area}\;\mathrm{between}\;\mathrm{day}\;0\;\mathrm{and}\;\mathrm{day}\;4}{Section}$$

The average migration rate per day was assessed by dividing migration distance by four.

On days 1, 3, and 5 after the seeding, the cell proliferation was assessed by using cell counting kit-8 (CCK-8, Dojindo Laboratories, Shanghai, China). PDMS patterns including flat surface were cut with a 6-mm biopsy punch and placed in 96-well plate. After being coated with fibronectin, the cells were seeded and incubated for 24 h. Then, 10 µL of CCK-8 solution was added to each well and the plate was put into an incubator for 4 h. The supernatant was removed from the plate and transferred to a new 96-well plate in order to avoid interference of PDMS. The absorbance was measured by microplate reader at 450 nm.

### 2.4. Fluorescence Imaging

Samples were washed with phosphate-buffered saline (PBS) three times and fixed with 4% formaldehyde in PBS for 15 min and then permeabilized with 0.25% Triton X-100 in PBS for 10 min. Fixed cells were pre-incubated at room temperature with signal enhancer (Image-iT^TM^ FX signal enhancer, Invitrogen, Thermo Fisher Scientific, Waltham, Massachusetts, USA) to prevent non-specific background staining before immunostaining with a 1:400 dilution of primary antibody for human vinculin (Sigma Aldrich, Saint Louis, MO, USA) for an hour at room temperature. Alexa 546 goat anti-mouse secondary antibody (Invitrogen, Thermo Fisher Scientific) was treated at a 1:200 dilution for an hour. F-actin was stained with Alexa Fluor 488 phalloidin (Invitrogen, Thermo Fisher Scientific) for 30 min, and the samples were placed on a glass slide with the patterned side touching the surface, followed by nucleus staining and mounting with mounting solution with DAPI (4',6-diamidino-2-phenylindole Vector laboratories, Burlingame, CA, USA) for 15 min. Fluorescence images were taken using an optical microscope. (IX71, Olympus, Shinjuku, Tokyo).

### 2.5. Scanning Electronic Microscopy Images

Cells were fixed with 2.5% glutaraldehyde for 10 min and washed in 0.1 M cacodylate buffer solution three times for 10 min each. Post-fixation was performed with 1% osmium tetroxide (Sigma Aldrich, St. Louis, MO, USA) for 1 h, and then the cells were dehydrated in gradual concentrations of ethanol for 15 min in each concentration. Ethanol was replaced with isoamyl acetate, which was finally substituted by hexamethyldisilazane (Sigma Aldrich, St. Louis, MO, USA). The samples were coated with 2-nm-thick platinum and imaged in SEM (FEI, Hillsboro, OR, USA) microscopy.

### 2.6. Statistical Analysis

For statistical analysis of the results, one-way analysis of variance (ANOVA) was used to evaluate their statistical significance. All data was obtained by at least three samples from a group and shown as mean ± SD (standard deviation). A *p*-value below 0.05 was considered statistically significant.

## 3. Results

### 3.1. Microgroove Patterns Affect Cell Shape and Orientation

**Figure 3:**
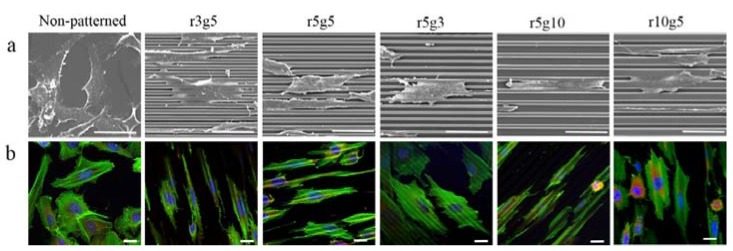
(**a**) Scanning electronic microscopy and (**b**) Immunofluorescence images of B-3 on various surfaces. Cells on r3g5, r5g5, r5g3. r5g10, r10g5, and non-patterned surface acted as control. Blue, Green, and Red fluorescence represents nucleus, F-actin, and vinculin, respectively. Scale bars indicate (**a**) 50 µm and (**b**) 20 µm.

**Figure 4:**
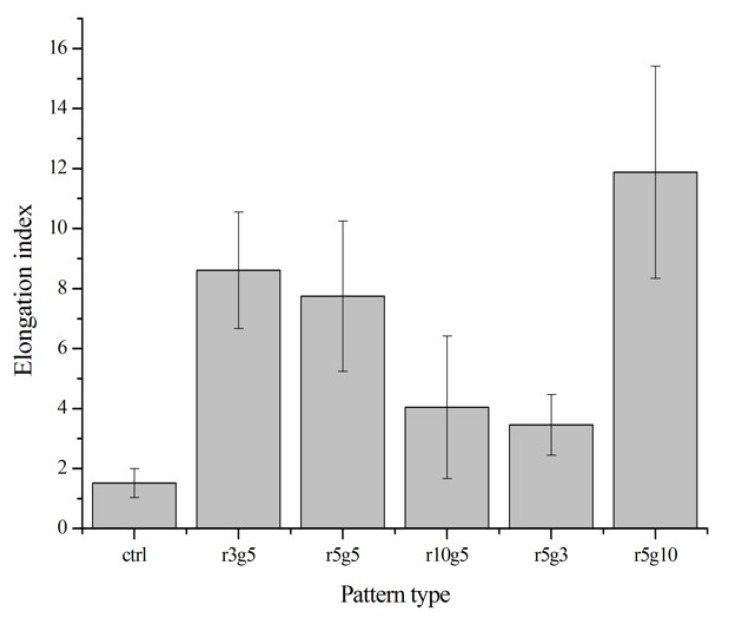
Elongation index. Elongation parameter is evaluated by dividing cell length into width. The different values indicate circular or linear cell shape (n = 10). Data expressed as mean ±SD. *p* < 0.001 versus control.

On the contrary to the cells on non-patterned surface, cells seeded on microgroove patterned surfaces had slender and longer shapes. In patterned groups LECs showed specific preference to orientation; in contrast, the cells in the non-patterned group were randomly located on the surface with no preference of orientation (Figure 3a,b). Cells in the patterned groups covered several ridge/groove units, with a single cell on a couple of ridges and grooves, except in r5g10 group cells which appeared to settle in the grooves. Elongation index (Length/Width) was assessed to compare the degree of elongation of cells on different patterns. Cells in patterned groups had higher elongation index than the cells in control group, which was approximately 1. This indicates that cells on patterns had the tendency to take longer shape, whereas the shape of cells on smooth surface was close to circular. Of all the patterns, the r5g10 group showed the highest elongation index, which means the B-3 on r5g10 pattern had the longest and narrowest cell shape (Figure 4). According to the elongation index, the cell shape took a more rounded form as the ridge width increased. On the other hand, the cells took a longer shape as the width of the grooves increased.

### 3.2. The Arrangement of F-Actin Depends on the Type of Patterns

Vinculin and F-actin were stained in order to observe cytoskeletal arrangement. In the patterned group, actin filaments in cells had the tendency to align in the direction of the groove patterns, in contrast to the randomly formed actin filaments on non-patterned surface (Figure 3a,b). Although the cells appeared to be aligned along the grooves on the surfaces of 5 µm groove width, in closer view the actin fibers were stretched across the patterns and small filopodia were protruding outwardly, regardless of the direction of the pattern. In the r3g5 group, cells had a retracted shape compared to other patterns, and short filopodia were reaching across the ridges. On r5g5 patterns, F-actin in most cells stretched along the grooves, but there were cells for which F-actin sprawled over the grooves and ridges. Although the cells on the r10g5 pattern seemed to align with the grooves, they spread wider than the cells in group with 5 µm groove width, and in most cells F-actin was formed at an angle to the grooves. Cells in the r5g3 group also showed the F-actin arrangement forming at an angle with no particular preference in the direction (Figure 3b). The r5g10 pattern showed the highest degree of arrangement of F-actin, with the majority of the cell body including nucleus confined to the grooves (Figure 3b).

### 3.3. Microgroove Patterns Interfere the Perpendicular Migration of LECs

**Figure 5:**
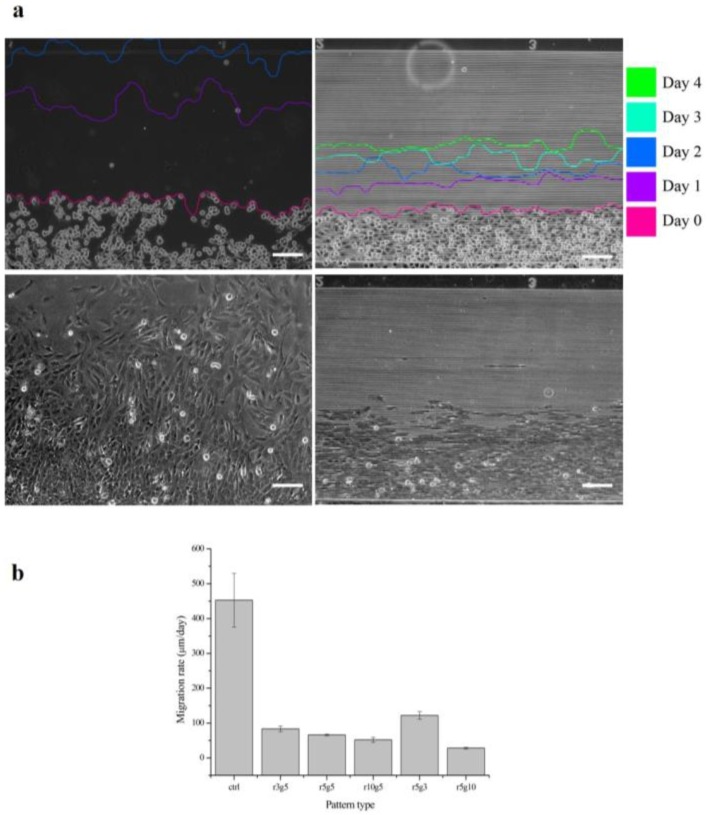
Migration rates of B-3 for 4 days. (**a**) The cell migrations at different days are traced. Control group of the cell migration rate (left) and r3g5 group (right) from day 0 to day 4 and the cell migrations on day 2 (bottom), respectively. (**b**) The cell migration rates of B-3 on different types of pattern. Scale bars indicate 100 µm. Data represent the mean ±SD of three independent experiments. *p* < 0.001 versus control.

Figure 5 shows the progression of collective cell migration on smooth surface and r3g5 patterned surface over the course of 4 days. After the PDMS piece that had been blocking the path was removed, B-3 started to migrate toward the empty space. The distance the cells migrated on the smooth surface for 1 day was longer than the distance of 4 days on the patterned surface. In all patterned group, cells migrating perpendicularly to microgroove patterns were slowed significantly compared to the cells on the non-patterned surface. All microgroove patterns the reduced migration rate of LECs by at least six times; for example, the average migration rate of LECs on the r5g10 pattern was 27.93062 μm/day, which is 14 times lower than the migration rate of cells in the control group (Figure 5a). Although every patterned surface had an effect of lowering cell migration rate, the r5g10 pattern was the most efficient surface in slowing down the cell migration. On the other hand, the r5g3 pattern was the least effective in inhibiting cell migration.

### 3.4. Proliferation Rate of Certain Pattern Represents Higher Migration Rate

**Figure 6:**
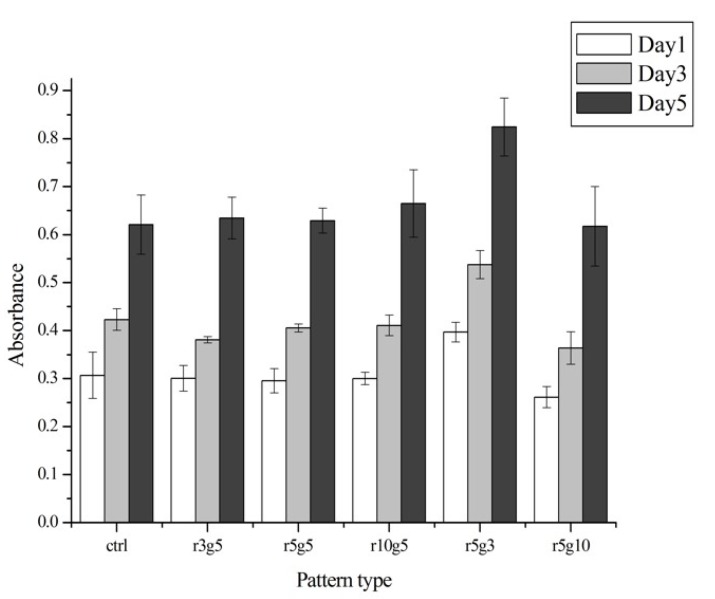
Cell proliferation rates of different surfaces on day 1 (white), day 3 (light gray), and day 5 (black) after the seeding. The absorbance was read at 450 nm by a spectrophotometer microplate reader. Data represent the mean ±SD of three independent experiments. *p* < 0.001 versus control.

In the proliferation assay, there was no significant difference between groups, except for the r5g3 pattern, which showed slightly higher proliferation rate than other groups from day 1 (Figure 6). The proliferation rate of the r5g3 group remained the highest throughout 5 days. The cell growth was similar in both patterned groups and the control group, suggesting that the microgroove patterns used in this experiment had no effect in terms of proliferation rate. Although the cells on all patterns seemed to induce cell stretching on the surface, it did not affect the cell viabilities, since proliferation rates in control showed no differences in the day 1, 3, and 5 (as shown in Figure 6). Therefore, we assumed that micro pattern-mediated cell stretching has negligible effects on cell viabilities. 

## 4. Discussion

Microgroove patterns were proven to be effective in suppressing collective human lens epithelial cell migration perpendicularly to the patterns. Though all of the patterns that were used were able to lower the collective cell migration rate of LECs, the 5 µm ridge/10 µm groove pattern was the most effective in slowing down migration. Jeon et al. showed the elongation and controlled movement of fibroblast on cross patterns and parallel stripe ridge patterns [18]. The LECs on the microgroove patterns showed elongated cellular morphology and aligned intracellular structures, such as F-actin and focal adhesions. Actin filaments are essential cellular components for the formation of cell shape and cell migration [19]. At the leading edge, crawling cells form a thin and broad-structured F-actin network called lamellipodia and long and narrow bundles of F-actin called filopodia [20]. Migrating cells protrude filopodia to sense the extracellular environment and extend lamellipodia to move forward [21]. Actin polymerization at the leading edge is the driving force by which the plasma membrane is pushed forward. Once the protruded membrane adhere to the surface creating new binding sites, actin filaments across the cytoplasm of the cell contract by F-actin–myosin II interaction, inducing translocation of the cell body while focal adhesions in the rear detach release from extracellular matrix [22,23,24,25]. Protrusion of leading edge and retraction of cell rear leads to the locomotion of migrating cells [26]. For a cell to move forward, the protruded lamellipodia must form nascent adhesions to the surface, which lead to maturation into focal adhesion. Disassembly of existing focal adhesion at the rear should be accompanied with the new adhesion formation for the translocation to be successful. The size of nascent adhesions is smaller than 0.2 μm, which grows to a size of 1–5 μm after they mature into focal adhesions [27,28]. Considering the size of focal adhesions, it is more difficult for the cells to create stable focal adhesions on ridges smaller than 5 μm in width than on patterns with wider ridges, and therefore the number of focal adhesions is likely to be smaller. On the contrary, wider groove width provides higher possibility for the nascent adhesion to develop focal adhesion, which will lead to translocation of cells. LECs on groove width less than 10 μm were not only unable to settle into the groove, but had less-organized morphology, and their lamellipodia were not as directionally controlled as the cells on patterns with 10 μm groove width (Figure 3a). Therefore, cells cultured on patterns with 10 µm groove width settled in the grooves and migrated along the pattern, rather than across the pattern. In other groups, most of the cells occupied several groove-ridge units and protruded lamellipodia perpendicular to the pattern, suggesting a greater tendency to cross the ridges and move across the patterns than cells in the groups with 10 µm groove widths (Figure 3a,b). While there were slight differences among pattern type, all patterns were capable of slowing down the migration of LECs. Especially, on the most effective pattern, the migration rate was 14 times lower than the smooth surface without any topographical features.

The lack of significant difference between patterns might be due to the fact that the patterns used in the experiment did not differ conspicuously in scale. Further study would have to be conducted to confirm the effect of pattern type on migration rate more closely. The lack of obvious tail structure that can usually be observed in motile cells is also one of the difficulties in determining the directional preference of cells.

Higher proliferation rate in the r5g3 pattern indicated the influence of surface topography on cell growth. Chaudhuri et al. showed that the proliferation of non-cancer epithelial cells was decreased on surfaces with microgratings, but not on surfaces with micropillars [29]. This could serve as a new approach to controlling LEC proliferation as well as migration rate to develop intraocular lenses to more effectively prevent PCO. Furthermore, the density of remaining lens epithelial cells after the cataract surgery is known to be significantly lower than the cell density used in this experiment since the epithelium is thoroughly removed; it is expected that the migration rate would be slower in vivo.

## 5. Conclusions

Posterior capsular opacification is the most common complication of cataract surgery. In this study we demonstrated the possibility of preventing PCO by applying surface topographical features to inhibit the migration of LECs. It appears that micrometer-scaled grooves on PDMS have the ability to make cells refrain from migration across grooves, acting as barrier to the cells and making them reluctant to crawl over ridges. Although all of the patterns we used had the same effect of slowing down the migration rate of LECs, the groove of 10 µm showed the best efficiency in interfering with migration. PCO remains as the most common obstacle of cataract surgery that causes the vision impairment process. Our study has shown that the migration of lens epithelial cells can be effectively restrained by microgroove patterns for attenuation of the development of PCO, therefore, applying microgroove patterns at the surface of intraocular lens materials is able to significantly decrease the incidence or prolongation of PCO. Apart from other treatments, a simple change in IOL design does not require any pharmaceutical agents or laser treatment, and has fewer side effects. Furthermore, if the cells can be confined to the patterned area long enough to progress towards the fibrosis, it is possible that the patients would not experience PCO at all. To ensure this, further studies need to be done, including in vivo test, and other pattern types can be provided to control the proliferation rate with the differentiation of LECs.
